# An Examination of Training Load, Match Activities, and Health Problems in Norwegian Youth Elite Handball Players Over One Competitive Season

**DOI:** 10.3389/fspor.2021.635103

**Published:** 2021-03-08

**Authors:** Christian T. Bjørndal, Lena K. Bache-Mathiesen, Siv Gjesdal, Christine H. Moseid, Grethe Myklebust, Live S. Luteberget

**Affiliations:** ^1^Department of Sport and Social Sciences, Norwegian School of Sport Sciences, Oslo, Norway; ^2^Norwegian Research Centre for Children and Youth Sport, Oslo, Norway; ^3^Department of Sports Medicine, Norwegian School of Sport Sciences, Oslo, Norway; ^4^Oslo Sports Trauma Research Centre, Oslo, Norway; ^5^Department of Physical Performance, Norwegian School of Sport Sciences, Oslo, Norway

**Keywords:** talent development, sport schools, youth sport, sports medicine, athlete development, injuries, illness, rating of perceived exertion of session (session RPE)

## Abstract

Talent development is integral to the policy and organizational practice of competitive sport, but has also been associated with excessive amounts of training and competition, and athlete injuries and illnesses. The lack of available prospective data on the training and match activities of youth athletes and their health problems is therefore of concern. The aim of this study was two-fold: (a) to examine the amount and frequency of training load, match activities, injury and illness incidence, and prevalence among Norwegian youth elite handball players over the course of the 2018–2019 competitive season; and (b) to explore whether the injury rates are related to the sex or competition level of players, or their membership of the youth international team. We recruited 205 handball players (64% female, 36% male), aged 15–18 years (17.2 years ± 0.9) from five different sport school programs in southeast Norway. Data were collected daily from September 2018 to May 2019, during the competitive handball season. The variables included types of athlete activities, the number of activities, the rating of perceived exertion (RPE), and the duration of training and matches. Injury and illness data were collected weekly using the Oslo Sports Trauma Research Center (OSTRC) questionnaire. The mean number of matches per week per player was 0.9 ± 1.29; the number of weekly training events was 6.1 ± 4.4; and the mean weekly session RPE was 986 ± 1 412 arbitrary units. The players reported a total of 472 injuries, and the mean number of injuries per player was 2.3 ± 2.9. The results demonstrated a 53% weekly injury prevalence, of which 38% were categorized as substantial injuries. Male players and players who participated at the highest level of senior competition and/or the youth international team reported significantly lower weekly incidences of health problems, compared to other players. Our findings showed that players enrolled in sport school programs are exposed to high training and competition loads, and that both general and substantial health problems are common. The potential implications for talent development and future research are discussed.

## Introduction

Talent identification and development are integral to the policy and organizational practice of competitive sport (Johnston et al., 2018). Typically, they are pyramidal in structure: at each successive stage, the number of available places for athletes decreases, and the amount of available support increases (Till and Baker, 2020). This “pyramid model,” associated with early and single sport specialization, can have negative consequences for athletes (Bailey and Collins, 2013). Medical practitioners have expressed concern that premature and excessive amounts of intense training and competition can, for instance, lead to higher injury rates, athlete burnout, or drop-out from sport at an early age (Bergeron et al., 2015). A few studies, however, have prospectively reported the training and match activities that characterize the pathways in systems of athlete development.

In Norway, researchers have begun to explore how athlete development systems and specialization pathways in sports such as handball affect the prevalence of injuries, and how overuse impacts youth athletes (Åsheim et al., 2018; Moseid et al., 2018). Athlete development in Norwegian handball emerges from an interplay between club-based practice and competition, sport academy secondary school programs, and the regional and national athlete development initiatives provided by the Norwegian Handball Federation. The model is loosely connected and decentralized, and is one in which emphasis is placed on providing practice opportunities for as many children and youth as possible. Regulations prohibit sport-specific specialization toward elite-oriented development before the age of 13, and financial sanctions can be imposed at the individual and club-level if the regulations are violated. The athlete development model is therefore structured in a way that facilitates later-age engagement without specialization (Bjørndal and Ronglan, 2020).

In Norwegian handball, challenges and opportunities arise in athlete development because players move regularly between practice and competitions in club, school, and federation-based settings (Bjørndal et al., 2015). When multiple actors—coaches and players, for example, are involved in both training and competitions at the same time, the complexity and intensity of practice activities increases (Bjørndal and Ronglan, 2018). As in other multi-centric models, this creates coordination challenges that need to be addressed in ways that ensure that athlete development is purposeful and negative impacts are minimized (Bjørndal and Ronglan, 2021). Research has shown that when the handball athlete development model is implemented successfully, complementary influences—such as being exposed to different coaches, players, competitive experiences, and training methodologies—can help to create diverse pathways to the elite level (Bjørndal et al., 2016).

Efforts to promote athlete and talent development in Norway have focused mostly on increasing opportunities for play and practice, often by adding more activities across the spectrum of activities offered by clubs, schools, and associations. These include, but are not limited to, the introduction of sport school programs at the lower secondary school level, as well as activities at the youth international team level, and age-based national club championship level (Bjørndal and Ronglan, 2020). Sport school programs can provide important opportunities for individually-focused and complementary training (Bjørndal and Ronglan, 2018; Bjørndal and Gjesdal, 2020). However, young athletes attending sport school programs may also experience many stressors, such as less sleep time, and significant increases in training volumes which may result in severe and long-lasting injuries (Kristiansen and Stensrud, 2016). Too much training and competition across clubs, schools, and federation settings can lead to injuries and burnout, and compromise the success of an athlete's transition to the adult elite level (Bjørndal et al., 2017; Kristiansen and Stensrud, 2020).

While the number of studies examining associations between training load and injuries in adult elite sport has increased in recent years (Griffin et al., 2020), no studies, to date, have simultaneously and prospectively reported on both injuries and training load, and match exposure among youth handball players, either in Norway or elsewhere. This is surprising given that training load and match exposure have been shown to be associated with overuse injuries (Soligard et al., 2016) and can impact adversely on athlete development in general (Myer et al., 2015). In the current research literature on training load quantification in handball, most studies have focused on load variables in match play or specific training drills (Buchheit et al., 2009; Michalsik and Aagaard, 2015; Luteberget and Spencer, 2017; Luteberget et al., 2018), and there is still little research on longitudinal training and competition load (Bresciani et al., 2010; Clemente et al., 2019). As such, the present findings present novel data on the training load of young handball players throughout a competitive season.

The aim of this study was therefore to explore the training load, number and frequency of match activities of athletes, and the incidence and prevalence of health problems (injury and illness) in Norwegian youth elite handball players over the course of a competitive season. Further, the study examines athlete health problems related to sex, competition level, and international team membership.

## Methods

### Design

The study was designed as a prospective exploratory study of training load, match activities, and injuries in Norwegian youth elite handball players over a competitive season. Athletes from five different sport school programs in southeastern Norway were invited, from the counties of Oslo, Viken, and Innlandet. A total of 231 players were asked to participate: 228 accepted the invitation, and 205 players consented and responded to the survey at baseline. The participating athletes were between 15 and 18 years old (17.2 years ± 0.9), 64% were female and 36% male.

### Ethical Statements

The study was approved by the Norwegian Centre for Research Data (reference number 407930) and the Ethics Review Board of the Norwegian School of Sport Sciences. The study followed ethical principles in accordance with the Declaration of Helsinki (Malik and Foster, 2016). All participants were provided both verbal and written information about the study, and informed consent was submitted electronically. Ethics approval did not require parental consent because all the players were above the age of 15 years. Participants were assured that their responses would be available only to the research team, that their responses would not be given to their coaches or respective sport schools, that their participation was voluntary, and that their consent could be withdrawn at any time.

### Data Collection

The data were collected longitudinally over 237 days in Norway during the competitive handball season, from September 2018 to May 2019. Data were collected via the Briteback AB online survey platform (https://lynes.io/).

Data related to player characteristics, their sport backgrounds, and their past experiences with practice and competition were collected at baseline. Players reported daily how many training sessions and matches they had attended, and the duration of these sessions in minutes. Each athlete reported his or her Rating of Perceived Exertion (RPE) using a modified Borg CR-10 scale (Borg et al., 1987; Foster et al., 2001) with integers and verbal anchors. Session RPE (sRPE) was calculated by multiplying the RPE of the players by the duration (in minutes) of the matches/training sessions (Foster et al., 2001). In instances in which players reported multiple trainings and/or matches on the same day, the sRPE was calculated for each session separately.

During the study, each of the 205 participating players was asked to provide a report on his or her training activity, on each of the 237 study days. The maximum potential number of replies was therefore 205 × 237 = 48,585 replies, and 17,268 replies (36%) were received (see Supplementary Table 1 for a discussion of the missing data). The replies (53%) were submitted on the same day when the players received the reminders (Supplementary Table 1).

The players also submitted daily reports on whether they had experienced an injury or illness since their previous response. Players were asked to record their health status by selecting one of three options: (1) no health problem, (2) new health problem, (3) worsening of an existing health problem. Players who selected either (2) or (3) were asked whether their health problem was an injury or an illness; if they reported an injury, players were asked to indicate whether it was an acute or overuse injury. Players were asked to report all health complaints, regardless of whether or not these impacted on their sport participation or whether they needed to seek medical attention.

Overuse injuries are difficult to capture via daily responses. For this reason, the OSTRC questionnaire was also used for the weekly self-reporting of health problems (Clarsen et al., 2013, 2014). The questionnaire was sent to every participant each Sunday morning, and a reminder was sent twice the following day if a response had not yet been received. A total of 1,479 OSTRC questionnaires were sent, and 97% were answered.

### Statistical Analysis

To examine the general load level of the study population, the weekly mean, standard deviation (SD), median, and maximum values of the following load characteristics were calculated:

- Number of matches
- Number of training sessions
- Number of free days (days without matches or training)
- Sum of minutes in activity
- Sum of the sRPE.

To visualize the group sizes, the percentage of players participating at each level of competition was plotted in a bar graph. The category levels were Premier League, Division 1, Division 2, Division 3, Division 4 or lower, Under-18, Under-16, and None. Similarly, player development levels were also plotted (Youth International Team, Regional Team, Player Development, and None).

Days on which players were unable to train due to injury (determined by their responses to the weekly OSTRC questionnaire) were excluded from these analyses. Vacation weeks and other weeks in which players did not have training or matches were considered to be part of the total load exposure for the players and, therefore, included. When calculating the weekly sum of minutes and the sum of the sRPE of player activity, only weeks missing two or fewer days were included per player. If a player's response indicated that he or she had no training or match on a given day, the sRPE and minutes were set to 0 for that day.

Daily injury incidence was calculated as the number of health problem incidents divided by the number of replies submitted and reported as a percentage. More detailed insights were possible by stratifying the analysis by illness and injury (both acute and overuse).

The response rate to the weekly OSTRC questionnaire was high (97%): we therefore used these to calculate weekly injury incidence, in addition to our original aim of calculating weekly prevalence. Only responses to the first OSTRC question (“Have you had any difficulties participating in normal training and competition due to injury, illness, or other health problems during the past week?”) that were *not* “Full participation without health problems” were considered to be indicative of a health problem and included in our calculation. For injury incidences, only players who did *not* respond that they had had an injury the week before were included in the numerator. When calculating injury prevalence, all injury replies were included. The denominator included all replies received. Weekly incidence and prevalence were stratified using the same subgroups as the daily incidence.

Substantial health problems were defined as injury or illness that reduced training volumes or performance to a moderate extent or worse, or to a complete absence from sport. This categorization was identical to the methodology used in Moseid et al.'s (2018) cohort study of the prevalence and severity of health problems in Norwegian youth elite athletes.

All numeric analyses were stratified by sex, player competition level (Level 1 = Premier League + Division 1; Level 2 = Division 2 + Division 3; Level 3 = Other Levels) and whether the player was a member of the Norwegian Youth International Team or not. The highest level of play included players who participated in multiple levels of competition play.

Temporal changes in training and competition load were visualized using the mean of the weekly sRPE sum plotted over time. Similarly, when comparing the injury rate with the measurement of exposure, the weekly match percent (the number of matches divided by the number of non-missing activity responses) was plotted against the weekly injury incidence (for comparability, daily data were used).

The data quality of the daily training load responses was deemed too poor to test for group differences. Group differences in injury frequency could, however, be tested using the responses to the weekly OSTRC questionnaire. To investigate whether the injury frequency was different between players who participated at different levels of competition play, traditional Chi-squared tests could not be used because it was not possible to assume that players could participate at one level of play only. In this instance, a Poisson regression was applied instead, and the response variable was the weekly health problem incidence. Two models were considered to assess if group differences were altered by injury definition: one using all health problems, the other using substantial health problems only.

Instead of choosing the highest participation level per player, as we did in the descriptive analyses, three logical variables were used. These were the number of players participating at: (a) Level 1, Premier League, or Division 1 (yes/no); (b) Level 2, Division 2, or Division 3 (yes/no); and (c) Level 3, Other Divisions and Levels (yes/no). By including all three variables in the model, we were able to adjust for instances in which players had participated at more than one level. Similarly, two variables were created for players who participated in: (a) an international team (yes/no), and/or (b) a regional team or no team (yes/no). In all the variable categories listed above, the “no” answers were included in the reference group.

The three levels of competition play (Level 1, Level 2, and Level 3) and the three team levels (International Team, Other Teams, or No Team) captured the same dimension, namely the level of play, and the risk of multicolinearity was therefore high. We thus modeled these two measures independently. When combined with the two injury definitions, this resulted in a total of four models.

The variable which described whether a player participated in a regional team or no team (yes/no) had a high multicolinearity with the variable describing participation in the international team. We therefore removed the regional or no team variable from the model and included only the variable for international team participation.

The following variables were included to adjust for potential confounding factors in the models: sex (male reference vs. female), age (≤ 16 reference vs. 17 and vs. ≥18 years), and time (study week). Coefficient estimates were transformed to Incidence Rate Ratios (IRR). We presumed it to be unlikely that there would be a linear relationship between time and injury frequency, and modeled this using Restricted Cubic Splines with three knots (Durrleman and Simon, 1989). The levels of overdispersion were checked, and robust standard errors calculated. The alpha level was set to 0.05. R scripts and data are available for reproducibility.

All analyses were performed using the software R version 4.0.2 with RStudio. version 1.3.1056.

## Results

**Figure 1:**
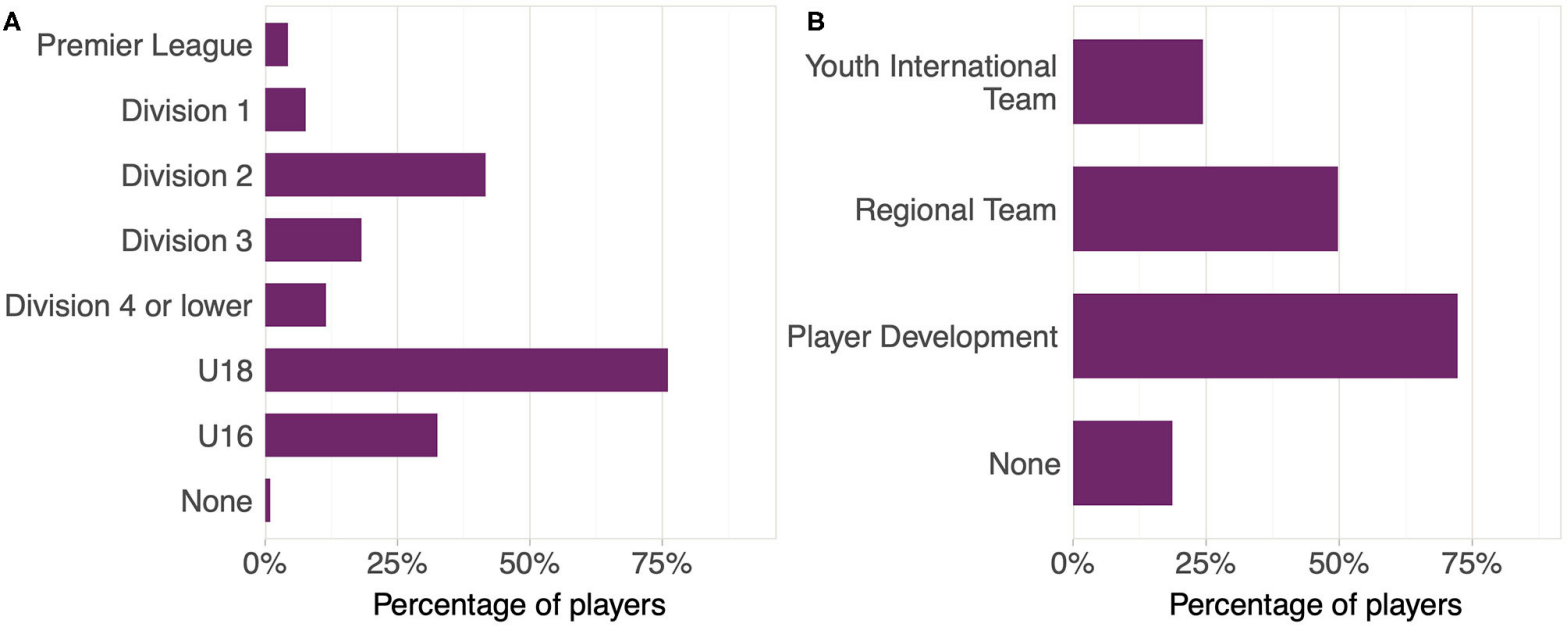
The percentage of the 205 handball players participating in different levels of **(A)** competition and practice play, and (**B**) experience from association-level player development activities. Players participating in more than one level could be counted in multiple categories (a player, for example, could be a member of both the Youth International Team and a Regional Team at the same time).

The characteristics of the players are reported in Table 1. Most players participated at the Under-18 competition level (76%), and at the Player Development level without being a member of the international team or a regional team (72%), and most players participated at multiple levels concurrently (73%, Figure 1). Both the number of matches, the number of trainings per week, the number of minutes in activity, and sRPE levels were highly variable in all groups (Table 2). The weekly training load remained high throughout the season, with the exception of the end-of-year holiday period and declined rapidly toward the end of the study (coincident with the end of the competitive handball season).

**Table 1:** Player characteristics.

**Characteristic**	***N* (%)**
Number of players	205
Sex	
Female	131 (64%)
Male	74 (36%)
Participate in additional sports	182 (88%)
Participate in multiple competition levels	153 (73%)
Participate in multiple teams	92 (44%)
	**Mean (SD)**
Age (years)	17.2 (0.9)
Age when starting handball (years)	7.0 (2.1)
Handball experience (years)	9.8 (2.6)
Number of other sports	2.0 (1.6)
Number of other levels	2.0 (1.0)
Number of other teams	1.7 (0.8)

**Table 2:** Weekly mean and median load parameters for handball players based on 21,737 trainings and matches.

		Sex^*^		Team^*^		Competition Level^*^		
	**Total (205)**	**M (74)**	**F (131)**	**Int. Team (50)**	**Other (155)**	**Level 1 (32)**	**Level 2 (89)**	**Level 3 (84)**
Number of matches								
Mean (SD)	0.9 (1.3)	1.1 (1.4)	1.0 (1.2)	1.2 (1.5)	1.0 (1.3)	1.2 (1.5)	1.0 (1.3)	0.9 (1.1)
Median (Max)	1 (14)	1 (14)	1 (10)	1 (14)	1 (13)	1 (14)	1 (13)	1 (7)
Number of trainings								
Mean (SD)	6.1 (4.5)	6.3 (4.5)	6.0 (4.5)	6.4 (4.7)	6.0 (4.4)	6.9 (5.0)	6.0 (4.1)	5.8 (4.6)
Median (Max)	6.1 (36)	6 (36)	5 (34)	6 (36)	5 (34)	7 (36)	6 (23)	5 (34)
Number of free days								
Mean (SD)	0.75 (1.3)	0.6 (1.2)	0.8 (1.3)	0.5 (1.2)	0.8 (1.4)	0.7 (1.4)	0.8 (1.3)	0.7 (1.3)
Median (Max)	0 (7)	0 (7)	0 (7)	0 (7)	0 (7)	0 (7)	0 (7)	0 (7)
Minutes in activity								
Mean (SD)	468 (207)	485 (206)	460 (207)	497 (206)	462 (207)	527 (197)	485 (194)	455 (191)
Median (Max)	480 (1,125)	510 (1,066)	465 (1,125)	518 (1,125)	480 (1,066)	555 (1,125)	495 (1,066)	450 (1,006)
Sum sRPE (AU)								
Mean (SD)	2,568 (1,229)	2,781 (1,338)	2,470 (1,163)	2,714 (1,270)	2,537 (1,219)	2,688 (1,163)	2,574 (1,204)	2,507 (1,251)
Median (Max)	2,565 (8,460)	2,730 (8,460)	2,505 (7,215)	2,730 (7,815)	2,535 (8,460)	2,730 (7,815)	2,565 (6,743)	2,520 (8,460)

*Stratified by sex, team (International Team, Other = Regional Team, Other, or No Team), and competition level (Level 1 = Premier League + Division 1; Level 2 = Division 2 + Division 3; Level 3 = Other Levels), with number of participants reported in parentheses*.

* *M, males; F, females; Int. Team, International Team*.

**Figure 2:**
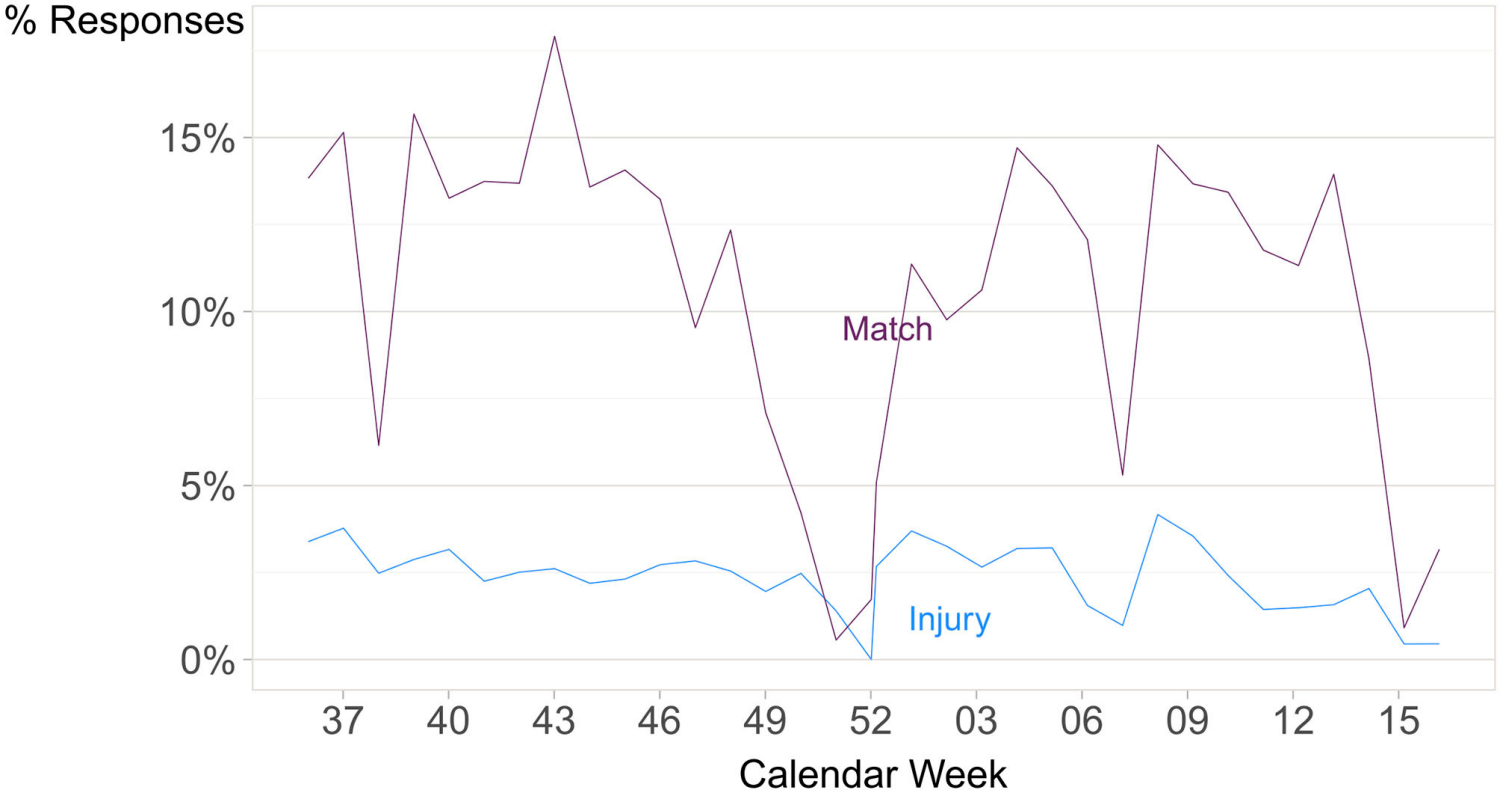
The weekly injury percentage compared to the match percentage across the season from week 37 in 2018 to week 17 in 2019. Based on 17 293 responses about player activity, and 17 379 responses about player injury from 205 handball players.

The players reported 472 injuries, with a mean of 2.3 ± 2.9 injuries per player during the study period. Daily injury incidence was 3% of the 17,379 player days (Supplementary Table 2). The weekly incidence of health problems was 12%, and players reported fewer illnesses than injuries, with no apparent differences between levels of acute and overuse injuries (Table 3). The weekly injury prevalence was 53% (Table 4). Males reported a lower weekly prevalence of health problems compared to females, consistent across all types of health problems (≈5–10% difference). The weekly injury percentage compared to the match percentage across the season is shown in Figure 2.

**Table 3:** Weekly incidence of health problems pertaining to the last 7 days for the whole study period.

		Sex^*^		Team^*^		Competition level^*^		
	**Total (205)**	**M (74)**	**F (131)**	**Int. T (50)**	**Other (155)**	**Level 1 (32)**	**Level 2 (89)**	**Level 3 (84)**
**All**								
Reponses^**^	1,447	446	1,001	263	1,184	219	684	544
Health Problems	176 (12%)	52 (12%)	124 (12%)	30 (11%)	146 (12%)	33 (15%)	82 (12%)	61 (11%)
Illnesses	49 (4%)	11 (2%)	38 (4%)	5 (2%)	44 (4%)	7 (3%)	25 (4%)	17 (3%)
Injuries	120 (8%)	38 (9%)	82 (8%)	25 (10%)	95 (8%)	26 (12%)	55 (8%)	39 (7%)
Acute	59 (4%)	21 (5%)	38 (4%)	14 (5%)	45 (4%)	18 (8%)	22 (3%)	19 (3%)
Overuse	61 (4%)	17 (4%)	44 (4%)	11 (4%)	40 (3%)	8 (4%)	33 (5%)	20 (4%)
**Substantial**								
Responses	1 446	446	1 000	263	1 183	219	684	543
Health Problems	119 (8%)	28 (6%)	91 (9%)	20 (8%)	99 (8%)	24 (11%)	52 (8%)	43 (8%)
Illnesses	38 (3%)	6 (1%)	32 (3%)	3 (0.2%)	35 (2%)	6 (0.4%)	18 (1%)	14 (1%)
Injuries	79 (5%)	21 (5%)	58 (6%)	17 (6%)	62 (5%)	18 (8%)	33 (5%)	28 (5%)
Acute	39 (3%)	11 (2%)	28 (3%)	11 (4%)	28 (2%)	13 (6%)	15 (2%)	11 (2%)
Overuse	40 (3%)	10 (2%)	30 (3%)	6 (2%)	34 (3%)	5 (0.3%)	18 (1%)	17 (1%)

*Stratified by sex, team (International team, Other = Regional Team, Other, or No Team), and competition level (Level 1 = Premier League + Division 1; Level 2 = Division 2 + Division 3; Level 3 = Other Levels)*.

* *M, males; F, females; Int. T, International Team*.

** *Seven responses indicated a health problem, but the type was missing*.

**Table 4:** Average weekly prevalence of health problems during the competitive season of all health problems and substantial problems, as well as subcategories of illness and injuries stratified by sex, team (International team, Other = Regional Team, Other or No Team), and competition level (Level 1 = premier league + Division 1; Level 2 = Division 2 + Division 3; Level 3 = Other levels).

		**Sex**		**Team**		**Competition level**		
	**Total (205)**	**M (74)**	**F (131)**	**Int. T (50)**	**Other (155)**	**Level 1 (32)**	**Level 2 (89)**	**Level 3 (84)**
Reponses	1,447	446	1,001	263	1,184	219	684	544
All Health Problems	760 (53%)	191 (43%)	569 (57%)	153 (58%)	607 (51%)	106 (48%)	351 (51%)	303 (56%)
Illnesses	134 (9%)	26 (6%)	108 (11%)	39 (15%)	95 (8%)	21 (10%)	65 (10%)	48 (9%)
Injuries	604 (42%)	155 (35%)	449 (45%)	110 (42%)	494 (42%)	78 (36%)	282 (41%)	244 (45%)
Acute	271 (19%)	60 (14%)	211 (21%)	58 (22%)	213 (18%)	60 (27%)	112 (16%)	99 (18%)
Overuse	333 (23%)	95 (21%)	238 (24%)	52 (20%)	281 (24%)	18 (8%)	170 (25%)	145 (27%)
Responses	1446	446	1000	263	1183	219	684	543
All Substantial Health Problems	546 (38%)	139 (31%)	407 (41%)	114 (43%)	432 (37%)	79 (36%)	268 (39%)	199 (37%)
Illnesses	102 (7%)	20 (5%)	82 (8%)	21 (8%)	81 (7%)	15 (7%)	50 (7%)	37 (7%)
Injuries	428 (29%)	115 (26%)	313 (31%)	87 (33%)	341 (29%)	58 (27%)	213 (31%)	157 (29%)
Acute	171 (12%)	31 (7%)	140 (14%)	42 (16%)	129 (11%)	42 (19%)	73 (11%)	56 (10%)
Overuse	257 (18%)	84 (19%)	173 (17%)	45 (17%)	212 (18%)	16 (7%)	140 (21%)	101 (19%)

*M, males; F, females; Int. T, International Team*.

The results from the Poisson regression models are presented in Table 5. In contrast to the unadjusted results shown in Table 3, players participating at the Level 1 competition level, Premier League, or Division 1, reported significantly fewer health problems than players who did not participate at Level 1 (IRR = 0.21, *p* < 0.001); these results were consistent for substantial health problems (IRR = 0.21, *p* < 0.001). Players who participated in the international team had significantly fewer health problems than players who did not participate in the international team (IRR = 0.15, *p* < 0.001), a finding also consistent for substantial injuries (IRR = 0.15, *p* < 0.001, Table 5). In addition, male players reported having significantly fewer health problems than female players (IRR = 0.47, *p* < 0.001) and this result was consistent for the reporting of substantial health problems among men and women (IRR = 0.34, *p* < 0.001). The covariates of sex, age, and time measured in weeks, demonstrated consistent results throughout all the models (Table 5).

**Table 5:** Incidence Rate Ratio (IRR), 95% Confidence Intervals, Robust Standard Error and *P*-value from four Poisson regression models to ascertain the association between different levels of play (Level 1 = Premier League + Division 1; Level 2 = Division 2 + Division 3; Level 3 = Other Levels) and weekly health problem frequency, and participating in the international team and weekly health problem frequency.

**Model**	Parameter^*^	**IRR**	**CI (lower to upper)**	**Robust SE**	***P*-value**
All Health Problems, Level	Intercept	2.63	0.9–7.5	1.41	0.071
	Level 1	0.21	0.1–0.3	0.05	<0.001
	Level 2	1.30	0.9–1.9	0.25	0.161
	Level 3	1.64	0.7–3.6	0.67	0.223
	Sex, Male	0.47	0.3–0.7	0.05	<0.001
	Sex, Female (Ref)	-	-	-	-
	Age ≥ 18	0.54	0.4–0.8	0.11	0.003
	Age 17	0.95	0.6–1.5	0.20	0.828
	Age ≤ 16 (Ref)	-	-	-	-
	Time (Week)	0.79	0.7–0.9	0.06	0.002
	Time (Week')	1.14	0.9–1.4	0.12	0.204
Substantial Health Problems, Level	Intercept	3.19	1.0–10.2	1.89	0.050
	Level 1	0.21	0.1–0.4	0.06	<0.001
	Level 2	1.28	0.8–2.0	0.28	0.267
	Level 3	1.04	0.4–2.4	0.45	0.926
	Sex, Male	0.34	0.2–0.6	0.08	<0.001
	Sex, Female (Ref)	-	-	-	-
	Age, ≥ 18	0.54	0.2–0.5	0.13	0.013
	Age, 17	0.80	0.5–1.3	0.20	0.380
	Age, ≤ 16 (Ref)	-	-	-	-
	Time (Week)	0.79	0.7–0.9	0.07	0.005
	Time (Week')	1.16	0.9–1.5	0.14	0.199
All Health Problems, Team	Intercept	9.78	6.3–15.0	2.12	<0.001
	Int. Team	0.15	0.1–0.2	0.03	<0.001
	Sex, Male	0.43	0.3–0.6	0.08	<0.001
	Sex, Female (Ref)	-	-	-	-
	Age ≥ 18	0.53	0.4–0.8	0.10	0.001
	Age 17	0.92	0.6–1.3	0.16	0.658
	Age ≤ 16 (Ref)	-	-	-	-
	Time (Week)	0.79	0.7–0.9	0.05	<0.001
	Time (Week')	1.15	0.9–1.4	0.11	0.156
Substantial Health Problems, Team	Intercept	7.54	4.8–11.8	1.72	<0.001
	Int. Team	0.15	0.1–0.2	0.04	<0.001
	Sex, Male	0.31	0.2–0.5	0.07	<0.001
	Sex, Female (Ref)	-	-	-	-
	Age ≥ 18	0.57	0.4–0.8	0.11	0.004
	Age 17	0.78	0.5–1.2	0.16	0.235
	Age ≤ 16 (Ref)	-	-	-	-
	Time (Week)	0.79	0.7–0.9	0.05	<0.001
	Time (Week')	1.17	1.0–1.4	0.12	0.130

*Time was modeled by Restricted Cubic Splines, and the IRR is therefore uninterpretable. Based on 176 health problems and 119 substantial health problems*.

* *Reference groups are as follows: Level 1 No Participation, Level 2 No Participation, Level 3 No Participation, Sex Female, Age ≤ 16 years, International Team No Participation*.

## Discussion

The aim of this study was to examine the amount and frequency of training load, match activities, and injury and illness incidence, and prevalence among Norwegian youth elite handball players over the course of the competitive season in 2018–2019. The study analyzed whether the rate of player injuries was related to the sex of the athletes, their competition level, or membership of the youth international team.

Our findings show that the study population experienced highly variable amounts of training and match activities across the competitive season, and that the incidence and prevalence of injuries and illnesses were high. The combination of higher exposure and the prevalence of injuries and illnesses could possibly compromise athlete development. Large inter-individual variations were also noted: training loads were highly variable between individuals, and female athletes experienced significantly more injuries compared to their male counterparts.

The sports histories of our study population were similar to those previously reported in studies of Norwegian players who have been active in youth international teams. Our study further indicates that athlete development pathways in Norwegian handball are characterized by diversification: a large number of the participants were involved in other sports in addition to handball. Furthermore, our results demonstrated that most of the players in our study (73%) participated at multiple competition levels and teams (44%). Similarly, Åsheim et al. (2018), noted that 72% of players aged 17–18 years participated in more than one handball team.

Other studies of youth athletes have reported higher volumes of training than those noted in ours: 9.3 (3.5) h per week of cross-country skiing (Landgraff and Hallén, 2020), for example, and 11.5 (4.2) weekly training hours in handball (Kristiansen and Stensrud, 2020). However, differences between the types of sports and in the methodologies used to analyze them (retrospective rather than prospective) could potentially explain such variations and highlight the potential importance of examining each sport separately and in consistent, comparative ways. In Spain, the number of training sessions per week during the competitive season in handball has previously been reported as between 4.0 and 5.3 for male handball players (Bresciani et al., 2010). One reason for the higher reported number of sessions in our study may have been due to the participants being enrolled in sport school programs. In contrast, the participants in the study by Bresciani et al. (2010) were above school age (20.1 [2.5] years) and therefore probably only training in club and/or association settings.

**Figure 3:**
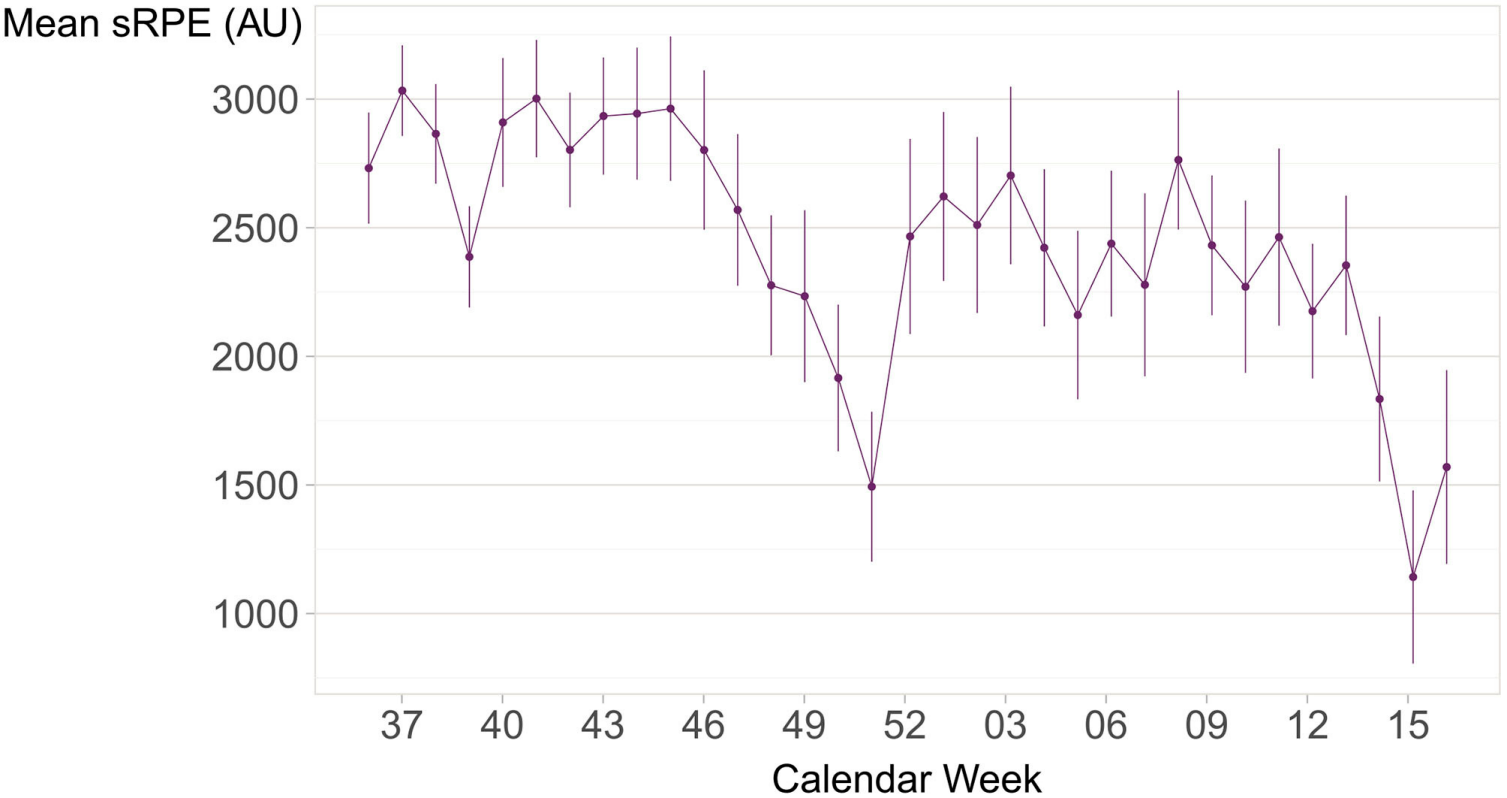
The mean of the sum session rating of perceived exertion (sRPE) value per player per week, with 95% confidence intervals, showing the change in the sRPE values across the season from week 37 in 2018 to week 17 in 2019. A total of 14,704 activity responses from 176 handball players were included.

Previous reports of sRPE in handball have reported sRPE values of 338–693 AU in training sessions in professional male handball players (Clemente et al., 2019), while female handball players have reported sRPE values of 443–630 AU in matches (Kniubaite et al., 2019). To our knowledge, only one previous study has reported weekly sRPE values in handball (Bresciani et al., 2010), ranging from 1,911 (200) to 2,712 (292) during the competitive season. Similar average values to ours were noted in the study, ranging from 1,500–3,000 AU (Figure 3). In basketball, differences in weekly sRPE load between playing levels have also been reported, with higher loadings reported among professional players compared to semi-professionals (Ferioli et al., 2018). In contrast, our data from Norway indicated that the sRPE loads were similar both at the team level (international vs. other) and the competition level—a reflection, possibly, of the unique and more democratic talent development pathways in the Norwegian sporting system which offers a broader base of players multiple opportunities and pathways within and outside the formal talent development pipeline (Bjørndal et al., 2015).

However, sport school participation cannot be disregarded as a key factor affecting the weekly load of youth handball players. As Bjørndal and Gjesdal (2020) have observed, sport school programs can help to bridge gaps in the training load between adult elite level players and/or youth international team players and others. In their study of the developmental paths of Norwegian female handball players, Bjørndal et al. (2016) found that the differences in the number of weekly training hours reported by athletes could be explained by whether the athletes were enrolled in a sport school program or not.

The *range* of training load noted in this current study is also of importance because such variability highlights the potential need for the individualization of processes to monitor training loads and training planning (Kiely, 2018). The maximum training load values (see Table 2) indicated that some of the players were exposed to very high weekly training loads, and a very high numbers of training sessions and matches. Training loads seemed to vary by setting: players reported higher sRPE scores when practicing in club settings compared to school or federation settings (see Supplementary Table 3), due possibly to the competitiveness of these club settings and the uniqueness of the sport school system in Norway, in which players do not compete for one particular school team (Bjørndal and Gjesdal, 2020). It could also be speculated that participation in multiple arenas could increase the risk of high training loads: as more coaches are involved, the demand for communication and coordination of training schedules will necessarily increase, potentially leaving only the individual players themselves with complete oversight over their total training load (Bjørndal and Ronglan, 2018). Further investigation is therefore needed into how training and competition load levels may vary depending on combinations of involvement in club, school and federation practice, and competition settings.

Our findings also indicated a high prevalence of injury and illness in the study population. These findings were similar to those reported in previous studies of Norwegian youth athletes: in specialized sport academy high schools, for example, the weekly prevalence of health problems was found to be 43% at any given time, and even higher among team sport athletes (Moseid et al., 2018, 2019a,b). Data from Swedish sport academy high schools (Von Rosen et al., 2018) and female youth elite athletes in team sport and gymnastics (Richardson et al., 2017) indicated similarly high levels of injury.

Female players were found to have significantly more injuries and substantial health problems. Sickness levels among female players, too, were higher compared to male players (11 vs. 6%). Previous research on youth elite athletes across different sports also reported that girls were at a higher risk of injury (Myklebust et al., 1997; Moseid et al., 2018). However, these variations may be due to differences in the way male and female players interpret symptoms, and may not necessarily indicate more serious underlying problems specific to female athletes in handball. It would be appropriate, therefore, to investigate how sport development programs should be structured to protect female athletes during their developmental years.

Surprisingly, the players who participated at the highest level of senior competition and/or the youth international team reported significantly fewer health problems (weekly incidence), compared to the others (IRR = 0.21, *p* < 0.001). One potential explanation for this may be that high-performing players represent the “survivors” of the athlete development system: being healthy is a prerequisite for transitioning successfully to the elite level (Bjørndal et al., 2017). Another plausible explanation may be that the best players are more valued and taken better care of by clubs and coaches (Bjørndal and Ronglan, 2018). Clubs may, for example, be incentivized to provide injury prevention programs at the highest level that are robust and well-implemented to protect players during the regular team practices.

Our study design did not allow us to test for causal relationships between the incidence of injuries and training, and competition loads. Instead, we aimed to establish an association between participation at particular levels of handball competition and health problems. We did not adjust for any measure of load levels (training load, match frequency, etc.). This would have been more appropriate if we had attempted to explain *why* players at certain levels of play had, or did not have, a higher frequency of health problems. Further, identifying a causal relationship would have been difficult due to apparent gaps in the load-related data we collected. However, when represented graphically, the incidence of match and health problems appeared to follow similar patterns: decreases/increases in the number of matches co-varied with decreases/increases in injuries, and appear to indicate that match exposure may be closely associated with incidences of injury. This suggests that an understanding of the association between training load and levels of injury is important to ensure that future talent development programs are developed and applied in sustainable ways. However, there is little consensus yet on how data on the frequency and volume of training and competition and injuries should be collected and analyzed (Bourdon et al., 2017; Bahr et al., 2020; West et al., 2020) and future research should focus on developing a deeper understanding of this apparent relationship.

### Strength and Weaknesses

Our study has methodological limitations. First, the amount of missing data may have led to selection bias in the data related to training load and daily incidence measures. Second, as the 2018–2019 handball season progressed, gaps in data reporting were observed. We used daily reporting in our longitudinal research to improve the quality of the data. However, the participants may have suffered from response fatigue and this could have potentially impacted on the quality of the data. However, the response rates to the weekly reporting of injury and illness were high and this suggests that the data are robust.

Despite these apparent limitations, the study also has several strengths. Our research is the first to provide a focused exploration of training and competition characteristics, in combination with an incidence and prevalence recording of health problems in youth handball players enrolled in school-based athlete development programs. Second, the prospective design of the study, which included a daily measurement of training load, provided a valuable starting point for quantifying and presenting a more detailed and nuanced picture of the daily activities of youth athletes, and how athletes develop over time. Third, the study provides insights into sub-group differences (related to sex, competition-level and youth national team membership).

### Practical Implications and Future Directions

The results of the current study indicate that handball players are subject to substantial training and competition loads and that levels of injury and health problems are high. This suggests that current training loads may not promote talent development or athlete well-being in sustainable ways. Our findings indicate that the key stakeholders involved must provide better ways of organizing the everyday activities of athletes, and of sustaining the quantity and quality of the training and competition needed. This may require a shift from the normative benchmarks used for young athletes at different ages and stages of sport development. The current monitoring of player activities and training loads may also be inadequate to the needs of athletes, given that athlete development is individually specific and can be non-linear.

More research is needed to provide insights into talent development programs, how to optimize training and competition loads, and reduce the injuries and illnesses associated with the training of elite youth athletes. It is likely that moving beyond singular perspectives, such as those rooted in physiology only, may aid in this process (Denison and Mills, 2014, p. 14). Examples include athlete-centered approaches to development and performance preparation in team sports (Woods et al., 2020). Intervention-based and more practitioner-driven research methods such as action research may be especially useful ways to combine inter-disciplinary, contextual, and practical perspectives.

## Conclusion

Our findings show that players enrolled in sport school programs are exposed to high training and competition loads, and that both general and substantial health problems are common. In our opinion, these forms of talent development are unsustainable. Efforts to strengthen athlete development should be based on a detailed knowledge of the impacts of training and competition loads and the injury risks within specific contexts. Our study represents a new step in the process of knowledge development.

## Data Availability Statement

The original contributions presented in the study are included in the article/Supplementary Material, further inquiries can be directed to the corresponding author/s.

## Ethics Statement

The studies involving human participants were reviewed and approved by Norwegian Centre for Research Data Ethical Review Board of the Norwegian School of Sport Sciences. Written informed consent from the participants' legal guardian/next of kin was not required to participate in this study in accordance with the national legislation and the institutional requirements.

## Author Contributions

CB and LL conceived and designed the analysis, collected the data, contributed to data analysis, and wrote the paper. LB-M performed the analysis and wrote the paper. SG conceived and designed the analysis, contributed to data analysis, and wrote the paper. CM and GM contributed to data analysis and wrote the paper. All authors contributed to the article and approved the submitted version.

## Conflict of Interest

The authors declare that the research was conducted in the absence of any commercial or financial relationships that could be construed as a potential conflict of interest.
