# Supplementary material for: An Examination of Training Load, Match Activities, and Health Problems in Norwegian Youth Elite Handball Players Over One Competitive Season
Source: Front Sports Act Living. 2021 Mar 8;3:635103. doi: 10.3389/fspor.2021.635103 (PMC7982662; doi:10.3389/fspor.2021.635103)
Supplement: Supplementary file 3 [file Data_Sheet_3.docx]

| **Table S1.** Data quality parameters. | | |
| --- | --- | --- |
| **Sample Size** | Number of athletes | 205 |
|  | Number of daily forms sent | 47 651 |
|  | Number of weekly OSTRC forms sent | 1 492 |
|  |  |  |
| **Missing data** | Missing daily load values | 30 383/47 651 (64%) |
|  | Missing daily injury observations | 30 272/47 651 (64%) |
|  | Missing weekly OSTRC form | 45/1492 (3%) |
|  |  |  |
| **Timeliness** |  |  |
| Daily form | Mean answering time, days | 0.7 (SD = 1.6) |
|  | Percentage forms answered on the same day | 53% |
|  | Max answering time, days | 119 |
| Weekly OSTRC | Mean answering time, days | 0.6 (SD = 0.9) |
|  | Percentage forms answered on the same day | 54% |
|  | Max answering time, days | 17 |

| **Table S2.** Incidence of daily reported health problems. Stratified by sex, team (International team, Other = Regional Team, Other or No Team), and competition level (Level 1 = Premier League + Division 1; Level 2 = Division 2 + Division 3; Level 3 = Other levels) | | | | | | | | | | |
| --- | --- | --- | --- | --- | --- | --- | --- | --- | --- | --- |
|  | | **Sex*** | |  | **Team*** | |  | **Competition level*** | | |
|  | **Total (205)** | **M (74)** | **Fe (131)** |  | **Int.Team (50)** | **Other (155)** |  | **Lv. 1 (32)** | **Lv. 2 (89)** | **Lv. 3 (84)** |
| Reponses(n) | 17 379 | 5 441 | 11 938 |  | 3 159 | 14 220 |  | 2 907 | 8 055 | 6 417 |
| Total** | 808 (5%) | 234 (4%) | 574 (5%) |  | 150 (5%) | 658 (5%) |  | 117 (4%) | 390 (5%) | 301 (5%) |
| Illness | 250 (1%) | 65 (1%) | 185 (2%) |  | 31 (1%) | 219 (2%) |  | 23 (1%) | 129 (2%) | 98 (2%) |
| Injury | 472 (3%) | 149 (3%) | 323 (3%) |  | 101 (3%) | 371 (3%) |  | 80 (3%) | 218 (3%) | 174 (3%) |
| Other | 85 (0.5%) | 20 (0.4%) | 65 (0.5%) |  | 17 (1%) | 68 (1%) |  | 14 (1%) | 42 (1%) | 28 (<1%) |
| * M = Male, Fe = Female; Int. Team = International Team; Lv.1, Lv. 2, Lv. 3 = Level 1, Level 2, Level 3  **One response indicated that a player had a health problem, but the exact type was not indicated | | | | | | | | | | |

| **Table S3.** The mean number of trainings, minutes in activity and sRPE each handball player has per week with their club, school environment, federation, or other (which includes self-training), based on 13 304 reported trainings from 205 players. | | | | | | |
| --- | --- | --- | --- | --- | --- | --- |
|  |  |  | **Training Milieu** | | | |
|  | **Total (13 304)** |  | **Club (5939)** | **School (4396)** | **Federation (273)** | **Other (2696)** |
| Number of Trainings |  |  |  |  |  |  |
| Mean (SD) | 6.1 (4.5) |  | 3.86 (2.3) | 2.50 (2.0) | 2.97 (2.5) | 2.31 (1.8) |
| Median (Max) | 6.1 (36) |  | 4 (20) | 2 (20) | 2 (16) | 2 (12) |
| Minutes in Activity |  |  |  |  |  |  |
| Mean (SD) | 468 (207) |  | 235 (105) | 190 (101) | 168 (121) | 139 (107) |
| Median (Max) | 480 (1125) |  | 240 (630) | 180 (960) | 135 (975) | 105 (916) |
| sRPE |  |  |  |  |  |  |
| Mean (SD) | 2568 (1229) |  | 1297 (679) | 976 (900) | 969 (665) | 772 (619) |
| Median (Max) | 2565 (8460) |  | 1260 (5100) | 900 (5340) | 840 (4665) | 570 (5160) |
|  | | | | | | |
